# Event and Comment

**Published:** 1910-08-15

**Authors:** 


### DENTAL REGISTER.

**Vol. LXIV. August 15, 1910. No. 8.**

### EVENT AND COMMENT.

NATIONAL DENTAL MEETINGS AT DENVER:—The annual meetings of our National bodies were held in the City of Denver, beginning with the American Society of Orthodontists, July 13th, and closing with the National Faculties Association and National Board of Examiners, July 25th and 26th. In spite of the unusually hot weather and the great distances most delegates had to travel, the attendance was remarkably large. More than 1200 persons registered 900 of whom are practicing dentists. Forty-four States and Territories were represented. Nearly 150 new members were added to the rolls. Of course Colorado had the largest representation, 312, Kansas came next with 77, then Missouri with 53, Nebraska 49, Illinois 42, Iowa 38, New York 33, Ohio 31, Texas 29, California 26, and so on down to Rhode Island, 1. The Colorado dentists did everything possible to make their guests comfortable and happy in spite of the fiercely hot weather, which they claimed was unprecedented, and which lasted through the entire season of meetings. The City and mountains near by are certainly beautiful and most interesting, and every opportunity for sight seeing was fully utilized and programmed by the local committee. A trip to the mountains, lasting all day, was tendered the guests by the Colorado dentists, including a fine picnic luncheon on the top of the mountains, the Los Angeles dentists contributing ten boxes of elegant oranges to the occasion, nearly 800 persons took advantage of this trip, which was called the Switzerland Trail. So far as grand scenery is concerned it certainly deserves its name, although, because of the long continued drought, the beautiful green foliage of the Switzerland Alps was missing. On the return, however the rose tints on the variously colored rocks from the rays of the setting sun more than made good the lack of grass, foliage and evergreen colorings.

THE ORTHODONTIA MEETING:—This society does not usually meet in conjunction with the National Dental Association, but this year it was thought wise to depart from its custom for the sake of many of its members living in the far west who are ordinarily unable to attend its sessions because of the great travel required. The meeting was unusually well attended, and an excellent programme was presented with good facilities for lantern work and clinical exhibits. About thirty regular members were in attendance and many others interested in the work visited the sessions. It seems like a waste of resources to continue this society as an independent organization. It would seem that it should become an integral part of the National Dental Society. It was organized to accomplish a specific purpose, the development of modern method in treating malocclusion of the teeth, which it has so well done that to day no specialty of dentistry is so firmly established on a basis of uniform practice. Is it not now time the society should bring back to the National society the strength and skill which it has acquired that it may help in the general uplift and assist in the plan of unification so much talked about and desired and which at present seems only slowly materializing.

FRATERNITY MEETINGS:—Two of the Greek letter dental Fraternity organizations, Delta Sigma Delta and Psi Omega held their annual conventions on Monday, July 18th. The Delta Sigma Delta organization is the older, and has a large membership among the more prominent men in the profession. It held an all day session, with a banquet in the evening attended by over 200 members. Its affairs seem to be in a most prosperous condition. The Psi Omega fraternity is comparatively a new organization, but it has become one of the strongest, having chapters in all the important dental colleges of the country. Its leaders are among the most energetic and progressive younger men in the profession, and it is destined to become strong and useful if as wisely controlled in the future as in the past. Between eighty and ninety members were in attendance.

The place of the fraternity in College life has been more or less critical and problematic, in some schools the fraternities seem to have exerted a wholesome and elevating influence, while in others they have caused the faculties much trouble and have been severely condemned. In the profes-fession their influence has sometimes been inimical to the best interests because their power has been used for political purposes. But on the whole we believe the fraternity has a useful function, and if its work is kept within the legitimate sphere of the purpose for which it was organized, it will do much for the elevation of professional dignity and attainment of the most laudable standards.

NATIONAL FACULTIES ASSOCIATIONS AND EXAMINERS:—These three organizations met at Denver at different times; each one is endeavoring to raise the standards of dental and education by its own methods. The new Faculties Association of American Universities consists of six of the Dental Departments of strong Universities and is endeavoring to bring about a uniform admission standard and curriculum among its members by a unanimous agreement, rather than by legislation, the method of the Old National Association. It is hoped in the near future that these six schools at least will demonstrate the feasibility of maintaining a creditable standard without financial disaster, which seems to be the largest item in preventing all schools from coming immediately to an agreement that will meet the approval of the profession and the State Boards of Examiners. If this can be accomplished by any or all of these organizations working together or each in its own way, the public will receive the benefits and the profession the honor to which it is entitled. It is a hard problem and will need strenuous efforts by all to bring it about; but it is an end so sadly needed that no struggle that promises results should be avoided. Let us hope that from the several and combined efforts we shall emerge at no distant day with a complete system of education under which all can unite and work together.

THE NATIONAL DENTAL ASSOCIATION:—Outside of the programme and regular routine business, the question of most interest to everyone seemed to be the adoption of some new scheme of reorganization or amendment to the present constitution and by laws, which would result in a closer affiliation with the profession and a materially larger increase in the membership of the Association. The wonderful success both professionally and financially of the American Medical Association has led many to think the adoption of its plan would bring similar benefits to the dental profession. Those who have studied the matter most thoroughly seem inclined to believe that some modification at least of this plan will be necessary. As a consequence the question of reorganization, after mature deliberation by the Executive Council, was deferred to a special meeting to be called by that body sometime during the coming winter, when it is hoped that every State Society will send one or more representatives and that a plan may be devised that will meet with general approval and hearty cooperation which is essential to the success of any movement of such wide and diverse interests. This of course defers the beginning of the publication of the societie's Dental Journal which was to have begun in October. The next meeting will be held in Cleveland, the third week in July. This being a central place it is expected that it will be the largest attended meeting ever held by the society.

The Ohio dentists made a vigorous campaign and won out from Rochester, N. Y., by a considerable majority. The officers elected for this year, are:—President, Dr. E. S. Gaylord, New Haven, Conn.; Vice President, for the East, Dr. C. S. Butler, Buffalo; Vice President, for the South, Dr. J. W. David, Corsicana, Texas; Vice President, for the West, Dr. E. R. Warner, Denver; Corresponding Secretary, Dr. C. W. Rodgers, Dorchester, Mass.; Recording Secretary, Dr. H. C. Brown, Columbus, Ohio; Treasurer, Dr. A. R. Melendy, Knoxville, Tenn.

The papers were all of such high order that it would be difficult to make distinctions, we can not however refrain from mentioning the splendid paper of Dr. J. Leon Williams on Anatomical and Esthetic Prosthesis, which was universally commended. It certainly lifted to a most exalted position a subject which seems rapidly degenerating in the esteem the profession. This paper will be one of our classics and should be studied carefully by every practitioner of dentistry.

The clinics under the management of Dr. F. O. Hetrick were numerous, 155 on the programme, and many of them of a very high order. The facilities were good considering the large number to be provided. The space for the surgical clinics was not what it should have been at all; but it is exceedingly difficult to give such clinics at meetings of this sort and those in attendance will not go to the hospitals. It may therefore be questioned whether such clinics should be attempted. The exhibits were as usual, many and varied. The large Auditorium building supplied ample space, and the exhibitors vied with each other in making their displays as attractive as possible.

